# How can folklore build children’s imagination: A systematic literature review

**DOI:** 10.12688/f1000research.180332.1

**Published:** 2026-06-15

**Authors:** Hana Andriningrum, Ujang Sugara, Wiyun Philipus Tangkin, Andriyani Afliyanti Dua Lehan, Dyah Indraswati, Sisca Wulandari, Ratih Purnamasari, Fauzi Fadliansyah

**Affiliations:** 1 Elementary Teacher Education, Universitas Negeri Surabaya, Surabaya, East Java, Indonesia; 2 Elementary Education, Universitas Negeri Yogyakarta, Yogyakarta, Special Region of Yogyakarta, Indonesia; 3 Elementary Education, Universitas Negeri Jakarta, East Jakarta, Special Capital Region of Jakarta, Indonesia

**Keywords:** Folklore, imagination, children, education, elementary

## Abstract

Folklore is a cultural medium that transmits values, traditions, and collective knowledge across generations, and it is considered a powerful tool to stimulate children’s imagination as well as support their cognitive and socio-emotional development. This study aims to address the role of folklore in fostering children’s imagination, particularly within elementary school contexts. This study employed a systematic literature review following PRISMA guidelines, with relevant articles identified from Scopus and Web of Science databases using keywords related to folklore, imagination, and children. The selection process was conducted based on predefined inclusion and exclusion criteria, focusing on empirical studies published within the last 25 years, and the selected studies were analyzed using a qualitative synthesis approach. The findings reveal that folklore contributes significantly to the development of children’s imagination through narrative engagement, symbolic representation, and exploration of cultural contexts. Folklore-based storytelling encourages children to construct mental imagery, develop creative thinking, and enhance emotional understanding, while interactive storytelling activities promote active participation and deeper comprehension among learners. In conclusion, folklore serves as an effective pedagogical tool in enhancing children’s imagination in elementary education, and its integration into classroom practices not only supports creativity but also strengthens cultural awareness and meaningful learning experiences, suggesting that educators should incorporate folklore into instructional strategies to optimize students’ imaginative development.

## Introduction

Children’s imagination is essential to their cognitive, emotional, and personal development. Imagination can help children anticipate reality and explore counterfactual possibilities, which are important in problem solving and creative thinking ( Harris, 2021; Hoff, 2020). In addition, imagination in children can also facilitate emotional regulation and behavioural development. Imagination allows children to express unrealized desires and navigate social dynamics, which is important for their emotional and social development ( Grindheim, 2020). Based on this statement, children’s imagination is important in child development because imagination affects cognitive abilities such as creativity and critical thinking, supports understanding and emotional regulation, and encourages personal growth by helping identity formation and social skill development.

However, not all children are given the space to develop imagination skills. In communist or religious countries, children’s imagination is not valued, so children’s imagination tends to be low. In contrast, imagination is valued in more prosperous and more developed countries, so children have higher imagination skills ( Maksic & Pavlovic, 2008, 2009). In addition, current educational practices implemented in schools emphasize that children conform to the times and excel in other skills such as critical thinking, creativity, communication, and collaboration rather than developing their imagination skills ( Stuart, 2013). This indicates that current educational practices in schools inhibit imagination skills rather than nurturing them.

Teachers and prospective teachers need to study children’s imagination because imagination can be used in educational settings by integrating it into teaching methods. For example, teachers can use pedagogical methods that support independent learning, that trigger thinking, and tolerate ambiguity to foster children’s imagination ( Roppola & Whitington, 2014). One of the efforts to build children’s imagination is through storytelling and folkloric storytelling activities. Through folklore, children are encouraged to explore various creative expressions using multimodal media. Folklore indirectly improves their imaginative abilities ( Jones, Pimenta, & Cooke, 2023). In addition, folklore can stimulate the memory process and help children form concepts of thinking beyond their limits. It indirectly contributes to their social development and imagination ( Agbenyega, Tamakloe, & Klibthong, 2017).

Through the imagination contained in folklore, children are encouraged to think critically, imagine situations, and create new ideas ( Febriyana, Pradana, Rozak, & Susiolo, 2024). This is because folklore is inherently imaginative and creative, full of mythical creatures, magical elements, and heroic adventures. Exploring folklore can stimulate students’ imaginations, encouraging them to create their own stories, artworks, and performances inspired by folklore ( Monica & Soplantila, 2024).

Folklore plays an important role in developing imagination in primary school students. Folklore contributes to the formation of imaginative thinking and enrichment of speech, fosters love of country, understanding of one belonging to society, and fosters respect for national traditions ( Ergashevich, 2021). In folklore, it is through imagination that children imbue the meaning of stories and relate them to real-life situations. When children participate in folklore, they engage in shared or collective imagination and create new knowledge, as each child can be led into deeper thinking ( Agbenyega et al., 2017).

Folklore circulating in the community has valuable material that shapes children’s imagination. Shaping children’s imagination through folklore can be done through activities. Activities are critical in developing creative thinking and problem-solving skills; for example, children can role-play or even create imaginary friends. This can help children mentally practice social scenarios and understand the environment around them ( Woolley & Gilpin, 2020; Woolley & Tullos, 2008). On the other hand, folklore can also help children develop cognitive and affective imagination. Cognitive imagination involves creating representations of the world, while affective imagination allows children to express and process emotions through symbolic encounters. This dual development is important for holistic cognitive and emotional development ( Diachenko, 2011).

Based on these readings, it is known that folklore contributes to improving children’s imagination. However, these studies are still scattered in various contexts and approaches that have not been systematically integrated. Therefore, this study aims to address and synthesize existing scientific findings and explore more deeply the relationship between folklore and children’s imagination development.

## Methods

### Research design

This study utilized a Systematic Literature Review (SLR) to synthesise literature regarding the role of folklore in shaping students’ imagination. Systematic Literature Review (SLR) is a rigorous method for identifying, evaluating, and synthesizing existing research on a specific topic. It aims to provide a comprehensive overview of the literature, ensuring transparency and reproducibility in the process. The primary purpose of an SLR is to map the state-of-the-art in a particular field, identify gaps in knowledge, and suggest future research directions ( Fundoni, Porcu, & Melis, 2023). This research was designed to answer the Research Question (RQ):

RQ1: How have folklore forms been used in previous studies to stimulate or build children’s imagination?

RQ2: What strategies, including pedagogical approaches, methods, and/or techniques have been reported to be effective in using folklore to develop children’s imagination?

RQ3: What aspects of children’s imagination are influenced by the use of folklore according to previous studies?

RQ4: What research gaps are found in the international literature regarding the relationship between folklore and children’s imagination?

### Data-based and search strategy

We used two scholarly databases, Scopus and Web of Science, ensured thorough and reliable coverage. The most recent and pertinent decade of the role of folklore in shaping students’ imagination from 2000–2025 was the focus of the search. We used Boolean Operators (AND, OR NOT or AND NOT) to expand or specify searches that make it easier to determine the articles. The search was conducted using a combination of keywords.

(folklore OR “traditional stories” OR “folk tales” OR folktales OR myths OR mythology OR legends OR “oral tradition” OR “cultural narratives” OR “traditional narrative”) AND (child OR “primary school” OR “elementary school” OR “young learners”) AND (imagination OR “creative thinking” OR creativity OR “fantasy development” OR “imaginative capacity” OR “creative cognition”) in the Scopus and Web of Science database. The inclusion and exclusion criteria in this study can be seen in Table 1.

**Table 1.** Description of the inclusion and exclusion criteria of the article.

Criteria	Description
**Inclusion**	
Topic	Topics related to folklore and imagination
Range	Articles throughout 2000–2025
Publisher	Scopus and/or Web of Science
Subject	Students in primary school
Context	Folklore and imagination
Language	Article written in English
Type	Empirical studies with qualitative, quantitative, or both approaches
Transparation	Clarity of sample and research design methodology (data collection and data analysis must be explained)
**Exclusion**	
Topic	Topics not related to folklore and imagination
Range	Articles before 2000
Article Type	Article not accessible and not in English
Subject	Not an elementary school student
Method	Literature Review

### Screening process

**Figure 1.**
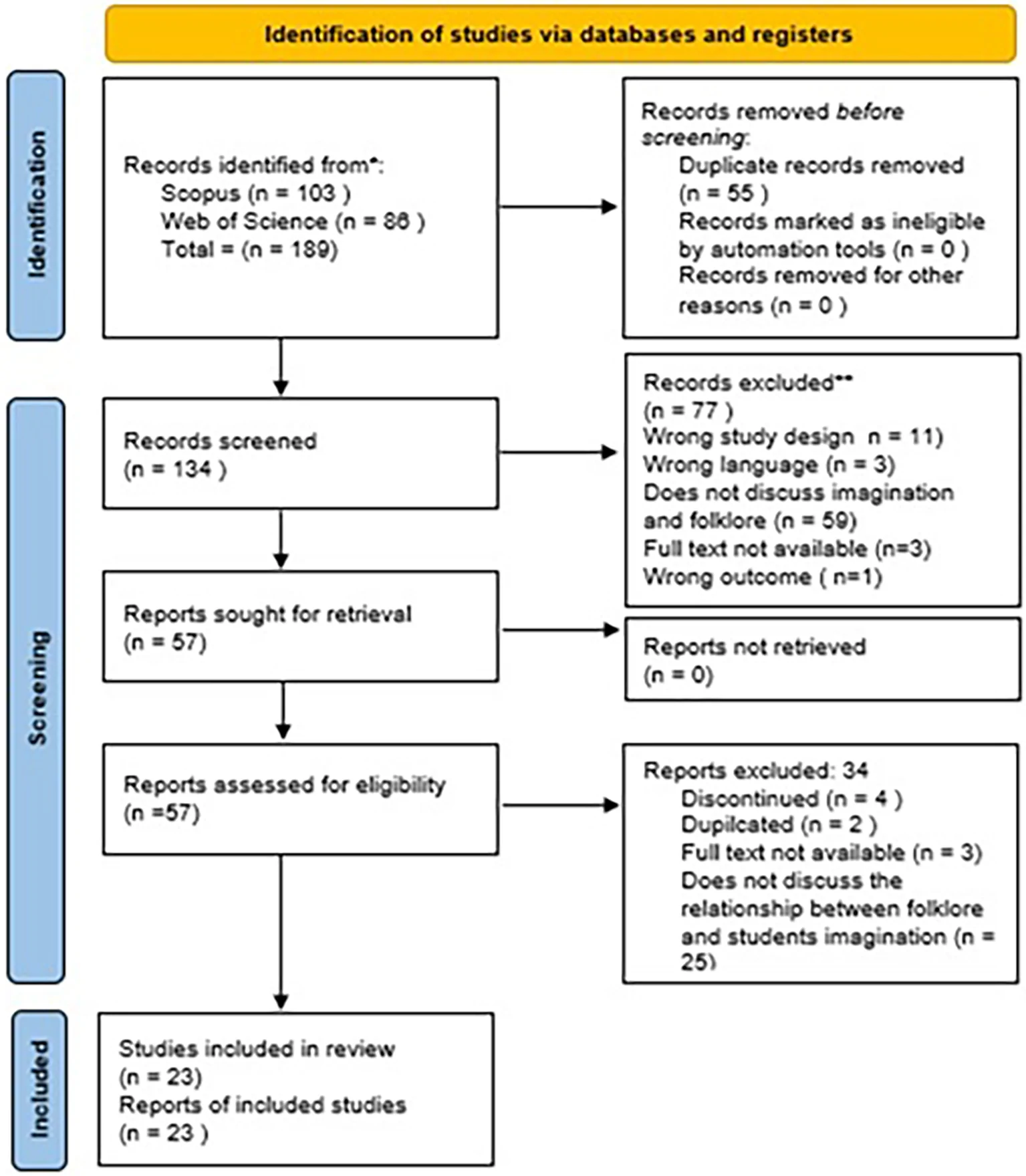
Preferred Reporting Items for Systematic Reviews and Meta-Analyses (PRISMA).

The article selection adhered to the PRISMA 2020 guidelines. Shown in Figure 1, 189 article titles were obtained from the Scopus and Web of Science database using the keyword combination (folklore OR “traditional stories” OR “folk tales” OR folktales OR myths OR mythology OR legends OR “oral tradition” OR “cultural narratives” OR “traditional narrative”) AND (child OR “primary school” OR “elementary school” OR “young learners”) AND (imagination OR “creative thinking” OR creativity OR “fantasy development” OR “imaginative capacity” OR “creative cognition”) on November 27, 2025, at 12.40 WIB (UTC + 07:00). Of the 189 article titles obtained, 55 titles were found to be duplicated across two databases, resulting in 134 titles. Of the 134 titles, 77 titles did not meet the inclusion criteria, such as not being in English, articles in the form of reviews, and book reviews. Therefore, the researcher excluded these 77 titles, leaving 57 titles that met the criteria. The researcher then reviewed these 57 titles to determine the suitability of the article’s topic and scope. This rigorous process ensured that only the most relevant and high-quality articles were included in the review. Of the 57 article titles, 25 articles were found not to address the relationship between folklore and elementary school students’ imagination. Furthermore, 9 titles could not be fully reviewed because they were discontinued, duplicated, and not available in journal databases, so the researcher did not select 34 articles in total for review. The researcher reviewed 23 articles on the relationship between folklore and elementary school students’ imagination. The comprehensive process is depicted in PRISMA flow diagram.

## Results

The following is a description of the articles discussed by the researcher, which includes the year of publication of the article, the name of the journal, and the rank of the journal that discusses the relationship of folklore to the imagination of elementary school students can be seen in Table 2. Meanwhile, the authors, country of origin, findings, aspects, contexts, and limitations of previous research can be observed in Table 3.

**Table 2.** Indexed journal publication.

Year	Name	Total	Rank	Cited
2015	International Journal of Children’s Spirituality		Q1	5
2022	International Journal of Early Childhood Special Education		Q4	0
2025	Folklor/Edebiyat		Q3	1
2023	Folklor/Edebiyat		Q3	1
2025	South Asian Review		Q1	0
2022	British Educational Research Journal		Q1	21
2024	English Academy Review		Q2	2
2022	Psychological Perspectives		Q4	4
2021	Integrative Psychological and Behavioral Science		Q1	10
2020	Interventions		Q1	7
2019	Folklore		Q2	12
2018	Revista Lusófona de Educação		Q4	48
2017	International Journal of Early Years Education		Q2	51
2017	Journal of Eastern Mediterranean Archaeology & Heritage Studies		Q2	1
2016	Africa		Q1	2
2016	Asian Social Science		Non Q	5
2014	Journal of Clinical Nursing		Q1	42
2010	Mental Health, Religion and Culture		Q2	42
2006	International Journal of Play Therapy		Q2	61
2003	European early childhood education research journal		Q2	64
2002	Literature and Theology		Q2	32
2002	Children’s Literature in Education		Q1	1
2004	Journal of Poetry Therapy		Q3	1

**Table 3.** Authors, Country of Origin, Year of Publication, Methods, and Findings.

Author	Country	Findings	Aspect	Context	Limitation
( Lee, 2015)	South Korea	Folklore serves as a tool to increase children’s sensitivity to the supernatural world. Folklore creates supernatural imaginations in children and leads them to higher realities, ultimately reaching divine truth.	Realist imagination and consolation imagination	An exploration of C.S. Lewis’s perspective on mythopoeia and fantasy literature as a means of ethical and spiritual formation for children	Focus on one character so that the perspective is limited to the values they uphold
( Bakhtiyorovna et al., 2022)	Uzbekistan	Folklore, especially the genre called “kyzykmachak” in Uzbek tradition, has a crucial role in enriching imagination, increasing curiosity, and providing aesthetic education to children.	Fantasy and myth imagination, visualization of environmental reality, logical thinking and humor	Exploring the history, genres, and poetics of Uzbek children’s folklore	The ambiguity of the boundaries of the folklore genre
( Kanik, 2025)	Turki	Folklore elements reinterpreted in popular culture products like cartoons play a crucial role in capturing the attention of young audiences and enriching their imaginations. Folklore cartoons take elements from fairy tales, legends, and myths and present them in a format that is familiar, engaging, and easily understood by children through character performances and narrative.	Creation of fantasy worlds, interaction of eclectic traditions and science, self-positioning and social structures, decision-making	Knowing the messages of food culture and the values of modern society are conveyed to children through reinterpretation of folklore elements	Children prone to misinformation, negative image
( Nurbekov & Salkynbay, 2023)	Kazakhstan	Fairy tales can stimulate children’s imaginations. Besides stimulating their imaginations, fairy tales also play a role in developing children’s behavior, speaking skills, language skills, and vocabulary. These fantastic stories are born from a belief in goodness and sincerity, and from the fantasy that victory in life always favors the good. These stories are easy to listen to and are enthusiastically embraced by children.	Understanding of the world, fantasy imagination, concepts of space and time, self and social reflection, character and values	A content analysis of Kazakhstani folklore on how simple and easy-to-understand fairy tales can convey profound cultural messages	Many children prefer watching fairy tales to reading them. This affects the depth of their cognitive interest in folklore
( Das, 2025)	India	Folklore serves as a medium for creating an intimate inner space between mother and child, protected from the rigid and rule-filled outside world (father’s world). Furthermore, folklore can also help children feel the presence of history and the ancient past, as if they were happening “here and now.”	Sensory and sonic imagination, spatial and territorial imagination, fantasy imagination, and emotional imagination	An exploration of Tagore’s thoughts on folklore as a means of building an imagination that transcends the rigid boundaries of colonial education	Gender bias due to a predominance of boys. There is a conflict between the interpretations of childhood by adults and the interpretations of children today
( Hajisoteriou et al., 2022)	USA	Folklore acts as a tool for “world-making” and “world-changing.” Folklore serves not only as entertainment, but as a means for children to understand the world, give meaning to their experiences, and connect with others. Specifically, folklore acts as a catalyst for social imagination, enabling children to imagine alternative societies that are more just than the current state.	Social imagination and creative and critical imagination	Workshop for children. Children engage in collaborative storytelling and critical dialogue about social justice issues	Not generalizable outside Cyprus, limited participants
( Gray, 2024)	South Africa	Ben Okri’s folklore work, “Every Leaf a Hallelujah,” plays a crucial role in transforming distorted beliefs about human control over nature and our separation from the natural world. Folklore serves as a means of ecological education, connecting human nature with Mother Nature through the power of creative imagination (Imaginatio Creatrix). This imagination is seen as a gateway to wisdom that underlies righteous action for living in harmony with the ecosystem.	Ecological imagination, intersubjective consciousness, mystical conjunction, inter-species consciousness, transcendence of space and time	a literary and ecocritical analysis of Sir Ben Okri’s first children’s book, Every Leaf a Hallelujah (2021)	Subjectivity of interpretation
( Howe, 2022)	UK	The Legend of Zelda is an online game based on mythology that shapes children’s experiences. For the case study subject, “R,” playing The Legend of Zelda series was a way to access mythology that felt relevant to him and a form of active imagination.	Active imagination, psychological imagination, visualization imagination of power	An exploration of the role of video games (particularly The Legend of Zelda series) as a form of “modern folklore” or new mythology that helps shape and develop children’s imagination and psychological state	Researcher bias, small scale of research
( Pinheiro & Simão, 2021)	Brazil	This research shows that children’s imagination develops through the interaction between collective myths and personal fantasies. Myths are considered discursive constructions inherited from other cultures and used by subjects when facing obstacles or challenges. Myths provide a framework for children to deal with everyday challenges and uncertainties. Children take meaning from cultural myths and integrate them into their personal developmental structures (phantasms), which then become imaginative patterns for acting and anticipating the future.	Flexibility, Plasticity, Malleability, Surplus of Vision	Exploration of comic strip works by elementary school students	Researchers cannot explore the entire research protocol and all the characters that appear in the data thoroughly
( Phalafala, 2020)	South Africa	Folklore, passed down from matrilineal family members to their children, is a primary knowledge system that informs and shapes Black intellectual culture and radical imagination. Folklore serves as a “primary archive” that restores a sense of community shattered by the colonial system. Through folklore, children are equipped with the wisdom to survive in the hostile environment of colonial modernity and apartheid.	Epistemic resistance, transnational identity	Exploration of folklore passed down through the maternal line (matriarchy) becomes the main foundation in forming identity and intellectuality	Focus on individual cases
( Hutton, 2019)	UK	Folklore with its “Wild Hunt” motif served as a paradigmatic element in shaping children’s and young adult fantasy literature in Britain in the late 20th century. Folklore provided rich mythological material and constructed creative narratives for young readers.	Primordial Natural Powers, Supernatural Visualization, Ambivalent Morality	This study uses the “Wild Hunt” motif as a paradigmatic example (primary model) for understanding how mythological material is reused in modern British children’s literature	This analysis is closely tied to a particular time period (the 1960s and 1970s) and a particular genre (teen fantasy), and acknowledges that the popularity of this motif may simply be a literary trend that waxes and wanes
( Oliveira & Cruz, 2018)	Portugal	This research uses African oral traditions combined with the philosophy of Ubuntu as a pedagogical tool to stimulate children’s imagination. Through folklore, children are encouraged to develop an understanding of other people, races, and religions, which in turn broadens the horizons of their social imagination.	Creatical Thinking (a combination of creative and reflective thinking)	Research on 24 4th grade elementary school students using a qualitative ethnographic approach by creating the “Kwesukasukela” project	The sample size and duration are limited, making it difficult to generalize due to its subjective nature
( Agbenyega et al., 2017)	Australia, USA, Thailand	Folklore serves as a pedagogical tool that allows children to create imaginary situations. Through stories, children not only listen but also engage in collective imagination, giving new meaning to animal characters (such as the personification of the spider Ananse) and connecting these fantasy situations to the realities of their social lives. Folklore positions children to engage in complex imaginative activities, going beyond the immediate parameters of the story.	Visualizing and Conceptualizing the Unknown, Relational Imagination, Meta-imaginary and Reflective Thinking	This study involved 23 children aged 5–6 years and two elders as storytellers using the stimulated recall method	Limited scale, differences in geographical context, risk of over-idealizing folklore
( Papadaki & Dakouri-Hild, 2017)	Greece & USA	Folklore plays a key role in shaping children’s constellations of ideas, perceptions, and beliefs about the past. Folklore, along with school knowledge and family contexts, provides a framework for children to interpret the physical archaeological remains they see around them. This allows them to imaginatively connect seemingly “lifeless” artifacts or ruins to more “living” and meaningful narratives of the past.	Topographic Visualization, Mythological Creature Visualization, Archaeological Space Visualization, Artifact Personification	This study aims to measure how local communities, especially primary school children, accept and engage with the past amidst a modern urban environment where ancient ruins often “collide” with everyday life	Children’s imaginations are heavily influenced by visual representations from museums or cultural animations, which sometimes obscure their original perception of the physical site. There is a distance between the “mythologized” past and the reality of rescue archaeology, which is often perceived by some communities as an obstacle to economic development or a disruption to daily activities
( Baynes-Rock, 2016)	Ethiopia	Baynes-Rock’s research provides an in-depth look at how folklore plays a crucial role in shaping children’s imagination and cognitive development regarding their relationships with animals, particularly hyenas (waraba). Folklore plays a role in constructing abstract concepts and fears, as a means of social control, and as a form of play for children.	Simple imagination related to Hyena, Anthropomorphism, Social Metaphor, Spatial Differentiation.	This study uses Paul Shepard’s theory that healthy human development depends on interactions with animals. It focuses primarily on the ontogeny of hyenas evolving from imaginary monsters in childhood to “social companions” (as Derma Sheikh conceptualizes) in adulthood	The theories used tend to be existential in nature, but they do not emphasize that imaginative play about animals is also a practical exercise for children to learn to avoid real dangers in their environment
( Majd & Bakhshaliyev, 2016)	Azerbaijan	Lullabies, as part of folklore, have a very high musical power that can carry children into a world beyond their real experience. This folklore helps children sleep with various kinds of dreams. In addition, mothers in Iran use skillful imagination in the song lyrics to describe their problems, worries, as well as their hopes and dreams, which indirectly stimulates children’s imagination.	Visualization of Nature and Abstract Values, Visualization of Roles and Characters	Qualitative analysis of Iranian folklore sung by mothers in various regions such as Khorasan, Fars, Kerman, and East Azerbaijan	Use of a single methodology, lack of resources during research, and difficulty in generalizing
( Van der Riet et al., 2014)	Australia & Thailand	Fairy Garden (FG) is a storyline or common thread centered on imagination. FG is also described as a space designed to stimulate children’s imaginations. Nurses read beautifully illustrated books about fairies, which helps spark their imaginations. Through FG, children can see and touch real objects inspired by the stories read to them, further encouraging the development of their imaginations.	Creativity through Symbols, Visualization and Meditation	This study explores the role of a healing environment called ‘Fairy Garden’ in a hospital in Thailand, where folklore elements are a key theme to stimulate the imagination of sick children	The findings are based on perspectives or stories from the nurses’ perspectives. Researchers note that these stories are not direct narratives from the children themselves, but rather interpretations of what the medical staff observed
( Walker, 2010)	UK	Folklore has the power to capture children’s imaginations because it often presents fantastical creatures, transformational experiences, or complex situations that allow children to immerse themselves in the story and connect it to their inner world. Harnessing children’s imaginations through folklore can be a powerful tool for providing transformative experiences on a psychic level, with positive impacts on emotional well-being. Mental health practitioners can reach children’s imaginations by exploring their memories of folklore. Folklore resonates strongly with the imagination, especially in children and adolescents who still possess a vibrant sense of wonder and creative potential.	Moral and Symbolic Imagination, Future Orientation	The context of this research is mental therapy for children and adolescents	There is no substantive evidence base (empirical evidence) that proves the efficacy of using fairy tales, myths, and legends in psychotherapeutic work
( Rubin & Livesay, 2006)	UK	Myths and the process of mythmaking form the basis of children’s fantasy realms, fantasy play, and the world of superheroes. Superheroes are seen as “contemporary mythological or folkloric icons” that hold a vital place in play because they help children confront real-world threats. The heroes or superheroes in these collective dreams and fantasies help children center and express their ideals, foster hopes and aspirations for the future, and provide an anchor connecting them to history.	Temporal fantasy, Meaning making	This research explores how superhero mythology can be used as a clinical tool to help children who have experienced multiple severe traumas	Lack of supporting empirical studies, gender and racial bias
( Lindqvist, 2003)	Sweden	Folklore, especially in the form of fairy tales, plays a crucial role as a foundational framework for children’s play. Research findings indicate that children’s imaginations are not tied to objects directly, but rather to narratives that give meaning to those objects and the actions they perform. Folklore narratives stimulate children to create and enter “play worlds” where they can act meaningfully.	Abstract Thinking Ability, Syncretic Creativity, Fantastic Hypotheticals	This research uses a cultural approach to play inspired by Vygotsky’s theory of the close relationship between play, art, and cultural awareness	Success depends on the role of adults
( Sky, 2002)	Norwegia	Fairy tales were considered valuable because they intensely engage the imagination. Romantics viewed fairy tales as essential childhood reading for fostering imagination. In England, Romantic thinkers even declared that fairy tales were the most appropriate reading material for children.	Spiritual and Visionary Imagination, Symbolic Imagination, Intuition and Subjective Feelings	This research highlights how fairy tales are used to normalize patriarchal ideologies, where women and children are often locked into assumptions of infantilism or a primitive closeness to nature	The fairy tales we know today have often been altered by upper-middle-class male writers to incorporate their own ideologies, thus losing the authentic voice of the folk oral tradition
( Bramwell, 2002)	UK	Myths serve as a source of inspiration that expresses imagination and awe. Myths are also closely related to other childlike qualities, such as imagination, delight, and wonder. The imagination of the child character, Kay Harker, is shaped by her knowledge of various popular story genres, which then serve as a framework for understanding the events she experiences.	Awe and Delighet, Self-consciousness, spiritual imagination	This research focuses on exploring the concept of “childness”, namely the quality of being a child that is dynamic, imaginative, experimental, interactive, and unstable	Research notes a strong nostalgic element from adult authors that may influence the way children’s worlds are portrayed (as idealized pasts)
( St Thomas & Johnson, 2004)	USA	Children use their imaginations to construct mythical structures that serve as symbolic spaces for healing and self-understanding. The stories and dramas they create are fairy-tale-like, possessing the transformative and communicative qualities of dreams at an archetypal level. In their play, children create a symbolically rich playscape that allows them to draw upon energies, characters, and representations from both animated and non-animated worlds.	Visual Thinking	This research focuses on play as an instrument of hope and a therapeutic tool for children	It should be noted that the content in this publication is the author’s opinion and its accuracy should be independently verified with primary sources of information

## Discussion

Imagination is a conscious and social act that is essential to life. Through imagination, children internalize the meaning of stories and relate them to real-life situations, engaging in complex imaginative activities ( Agbenyega et al., 2017). Folklore plays a multifaceted and crucial role in building children’s imaginations, influencing their cognitive, emotional, and social development. This role is emphasized through various genres such as myths, fantasy fiction, folktales, and oral traditions. Folklore, particularly myths and fantasy fiction (also called mythopoeia), has a powerful allure that influences children’s hearts ( Lee, 2015).

### The role of folklore is used to stimulate children’s imagination

Various forms of folklore can be utilized to stimulate children’s imaginations because folklore presents rich narratives, supernatural realities, and allows children to experiment with their understanding and emotions. When children read folklore or are read to by folklore, children will act as “sub-creators” or subordinate creators, where their minds participate in a “Secondary World” that has its own laws. Children’s participation in the “secondary world” encourages children to use “fantastic hypotheses” by asking “What would happen if…?” This process challenges their imaginations to solve problems in scenarios that go beyond their intellectual limitations and everyday experiences ( Lindqvist, 2003). What is interesting from this finding is that each form of folklore can stimulate different imaginations.

For example, folklore, such as folktales, often uses personified animal characters (such as the story of the Ananse spider) to help children develop moral and social concepts ( Agbenyega et al., 2017). Because these characters are not physically visible, children are forced to imagine them, conceptualize what they hear, and think about things they have never experienced. This activity stimulates their ability to think abstractly and develops “creative thinking,” or a combination of creative and reflective thinking to find future solutions ( Lindqvist, 2003; Oliveira & Cruz, 2018). This is the implication of folktales in stimulating social imagination.

Other findings also indicate that folklore can also serve as a medium or “stepping stone” for understanding others from different backgrounds ( Oliveira & Cruz, 2018; Phalafala, 2020). In other words, folklore plays a role in developing social imagination and empathy in children. By reading or being read to, children can position themselves as the characters in the folklore. Characters in folklore experience bitter and sweet lives which are also felt by children so that children can develop empathy when being read to or reading folklore ( Hajisoteriou, Panaou, & Angelides, 2022).

Furthermore, the imagination created when children read or are read to by folklore is social imagination. After reading or being read to by children, they will create creative and innovative “counter-stories.” The process of creating “counter-stories” begins with dissatisfaction with the plot or an unfair conflict resolution, creating a plot that is appropriate and just for the children ( Hajisoteriou et al., 2022). This aligns with the main concept of Bruner’s narrative theory. Bruner argues that narrative is a way of thinking that differs from paradigmatic thinking. Narratives help individuals organize their experiences and knowledge in meaningful ways, enabling them to construct and reconstruct their understanding ( Kang, 2017; Rutten & Soetaert, 2012).

Another form of folklore that can stimulate the musical imagination is lullabies, which are considered to be the primary form of music in verbal form. The musical power of lullabies, involving the use of rhythm, harmony, coherence, and rhyme, is so powerful that their melodies can transport children into an imaginary world beyond their physical experience ( Majd & Bakhshaliyev, 2016).

Furthermore, lullabies and folk songs also serve as a means of cultural transmission. The songs in lullabies and folk songs reflect the values, beliefs, and practices of a community, which are passed down from generation to generation ( Doja, 2014; Garrido & Davidson, 2019; Uǧurlu, 2014). These songs often contain elements of community history, myths, and social norms, which help children understand and internalize their cultural heritage. These experiences provide the foundation for stimulating children’s musical imagination later in life. One way to do this is by expanding their imagination of how sound and meaning can be expressed.

Overall, the role of folklore in children’s imagination can be likened to a magical compass; folklore not only guides children to explore vast and limitless fantasy worlds, but also provides a moral map and direction, helping them to navigate the complexities of reality, shape their own identities, and imagine the possibility of a better world ( Nurbekov & Salkynbay, 2023; St Thomas & Johnson, 2004).

### Effective strategies in shaping students’ imagination using folklore

Having established that folklore can stimulate children’s imagination, the next question that needs to be addressed is how to use folklore to stimulate children’s imagination. The author found numerous strategies that can be used in teaching folklore to children to stimulate their imagination. The first is the integration of read-aloud and manipulatives (physical objects or props) which are highly effective as a pedagogical and therapeutic approach ( Van der Riet, Jitsacorn, Junlapeeya, Dedkhard, & Thursby, 2014). This strategy leverages the power of oral narrative combined with sensory interactions to stimulate imagination, support healing, and build social connections in children. This strategy is considered successful because the combination of sound (auditory) and object manipulation (tactile) creates a superior multisensory learning environment.

The changes that occur in children are that they can “forget” the pain by shifting their focus to listening to read-alouds and manipulatives. Furthermore, they also become calmer because the manipulative objects used serve a spiritual function and become a means of meditation and prayer, providing spiritual peace. Overall, this strategy serves as a “light of hope” that allows children to let go of rigid, conscious thought patterns and freely express their feelings through imagination ( St Thomas & Johnson, 2004).

Another strategy involves using gamification to convey folklore to children. Gamification of folklore has proven effective in stimulating their imagination, as demonstrated by research from Oliveira and Cruz (2018) on the Kwesukasukela project. Gamified storytelling encourages students to become “creactical” thinkers, a combination of creative and reflective thinking, to produce original work and find solutions to future problems. Activities like creating a story map force the brain to work systematically to solve narrative problems using imagination.

### Aspects of children’s imagination influenced by the use of folklore

***Identity formation and self-reflection***

Children’s imagination, shaped through folklore, serves as a means for them to construct their ethical and spiritual identities ( Lee, 2015). Children’s imagination can be shaped through a process of reflection when reading or listening to folklore. This folklore leads to the formation of children’s self-awareness and the evaluation of their actions in real life ( Nurbekov & Salkynbay, 2023). This is illustrated in the Ghanaian folklore of Ananse. In this folklore, children are known to use their imagination to question their self-image and envision an “ideal self” they aspire to, such as being honest and selfless ( Agbenyega et al., 2017). Other folklore (lullabies) also contribute to the formation of positive feelings about their name and self-worth in children through imaginative words of maternal affection ( Majd & Bakhshaliyev, 2016).

***Social imagination and justice***

Folklore is an important tool for igniting social imagination. Social imagination is the ability to imagine a vision of a better or more just society ( Hajisoteriou et al., 2022). Social imagination can be fostered through collaborative storytelling. The benefits of collaborative storytelling for children include developing empathy and challenging stereotypes. First, it fosters empathy. When children listen to collaborative storytelling, they indirectly imagine the world through the eyes of others and experience the lives of others as if they were their own ( Hajisoteriou et al., 2022). Second, it challenges stereotypes. Some folklore contains stereotypes about race, gender, and social status. Children imagine deeply unfair stereotypes from these folklore stories. They then create “counter-stories” that build on their previous imaginations to challenge injustice ( Hajisoteriou et al., 2022).

***Modern mythology and active imagination***

In the modern context, imaginations formed through folklore have also penetrated new media such as video games (e.g., The Legend of Zelda) and superhero mythology ( Howe, 2022; Rubin & Livesay, 2006). This activity is seen as a form of active imagination (a Jungian concept) in which individuals consciously interact with archetypal content to aid in maturation and overcome childhood trauma ( Howe, 2022). Superheroes in children’s imaginations serve as contemporary icons that help them overcome feelings of helplessness in an adult-dominated world ( Rubin & Livesay, 2006).

### The gap between folklore and children’s imagination

***Lack of understanding of the relationship between folklore and complex thinking processes***

Although folklore is recognized as a pedagogical tool, further research is needed to understand in depth how folklore facilitates complex thinking and its specific role in child development. Current research methodologies are not yet considered definitive in explaining the mechanisms by which folklore-fueled imagination transforms into higher mental functions ( Agbenyega et al., 2017).

***Classification and incomplete theoretical relationships***

Special research is needed to create a complete genre classification in children’s folklore (such as in Uzbek children’s folklore) and to strengthen the theoretical foundations of the relationship between children’s folklore and adult folklore ( Bakhtiyorovna, Rakhmonovich, & Bakhtiyorovna, 2022). Furthermore, the evolutionary history of certain mythological motifs in children’s fantasy literature (such as the Wild Hunt motif ) still requires a broader review to understand the interaction between folklore and imagination ( Hutton, 2019).

***Generalization problems and research context***

Many studies on folklore and children’s imagination development are very local (for example, conducted in only one school in a city in Ghana). There is a research gap in comparing these results to other contexts, such as rural children, to determine whether the influence of folklore on children’s imagination development is universal or strongly influenced by the home environment ( Agbenyega et al., 2017).

## Conclusion

This study emphasizes folklore’s role in shaping elementary school students’ imagination through various dimensions. Folklore provides a rich narrative framework that allows children to explore complex relationships ranging from human relations with animals, ecological awareness, social dynamics, humour, to the construction of gender and the virtualization of cultural narratives. The imaginative processes shaped by folklore are not singular but contextualized and culturally rooted, allowing children to connect with local values while responding to contemporary realities. In addition, the recontextualization of folklore in the digital space shows the flexibility and relevance of folklore in encouraging children’s imagination. These findings emphasize the importance of integrating culturally responsive and critically reflective folklore to support children’s holistic cognitive and socio-emotional development.

However, the limitation of this study is the limited use of the keywords “folklore” or ‘folklorism’ and “imagination” in double database, there are Scopus and Web of Science, with the hope that the findings of the article focus on discussing the relationship between folklore and the imagination of elementary school-age children. Further research is needed to expand the findings on the link between folklore and children’s imagination by adding keywords such as “folktales” and “fantasy” with a broader range of years and more databases. This could open up opportunities to reveal the role of folklore in shaping children’s imagination specifically and in more depth.

Teachers and parents can integrate narratives in folklore into learning activities at school and at home, critically and contextually. Based on these findings, folklore can be utilized as a creative medium to develop narrative literacy, social empathy, and environmental awareness in children. However, teachers and parents should not necessarily choose folklore as a learning resource, emphasizing passing on culture. Teachers and parents can choose folklore that supports the values of equality, diversity, and inclusiveness.

These results also open space for education policymakers to regulate policies regarding local knowledge-based curricula responsive to the development of children’s imagination, which is rarely considered in learning activities in formal education institutions. Folklore as an intangible cultural heritage should receive more recognition, not just as part of cultural preservation, but should be emphasized as a potential learning resource. In addition, policymakers can ensure that narratives lifted from folklore have been critically reflected so as not to reproduce inequality and unrest, including social inequality, nature, and gender bias.

## Data Availability

No data associated with this article. Repository name: Data Systematic Literature Review Folklore https://doi.org/10.5281/zenodo.20034230 ( Sugara, 2026). Zenodo PRISMA_2020_checklist (2). https://doi.org/10.5281/zenodo.20034230 ( Sugara, 2026). Data are available under the terms of the Creative Commons Attribution 4.0 International license (CC-BY 4.0).

## References

[ref1] Agbenyega JS Tamakloe DE Klibthong S : Folklore epistemology: how does traditional folklore contribute to children’s thinking and concept development? *Int. J. Early Years Educ.* 2017;25(2):112–126. 10.1080/09669760.2017.1287062

[ref2] Bakhtiyorovna AN Rakhmonovich AB Bakhtiyorovna AN : The Essence and Genre Features of the Term “Kyzykmachak”. *Int. J. Early Child Spec. Educ.* 2022;14(3):2012–2023. 10.9756/INT-JECSE/V14I3.237

[ref3] Baynes-Rock M : THE ONTOGENY of HYENA REPRESENTATIONS among the HARARI PEOPLE of Ethiopia. *Africa.* 2016;86(2):288–304. 10.1017/S000197201600005X

[ref4] Bramwell P : Peter Bramwell Opening The Box of Delights. *Child. Lit. Educ.* 2002;33(2):117–129.

[ref5] Das N : Between Speaking and Writing: The Limits of Modernity in Tagore’s Poetry. *South Asian Rev.* 2025;0(0):1–15. 10.1080/02759527.2025.2483549

[ref6] Diachenko OM : On major developments in preschoolers’ imagination. *Int. J. Early Years Educ.* 2011;19(1):19–25. 10.1080/09669760.2011.570996

[ref7] Doja A : Socializing enchantment: A socio-anthropological approach to infant-directed singing, music education and cultural socialization. *Int. Rev. Aesthet. Sociol. Music.* 2014;45(1):115–147.

[ref8] Ergashevich AU : Folklore and It’s Role in The Development of Preschool Children. *Eur. Scholar J.* 2021;2(3):164–165.

[ref9] Febriyana I Pradana AE Rozak A : Exploration of Elementary School Students’ Imagination Through Children’s Stories: Creative Thinking Strategies. *International Journal of Linguistics, Literature and Translation (IJLLT).* 2024;7(11):168–175. 10.32996/ijllt

[ref10] Fundoni M Porcu L Melis G : Systematic literature review. *Researching and Analysing Business.* London: Routledge;2023; pp.55–72. 10.4324/9781003107774-5

[ref11] Garrido S Davidson JW : Childhood. *Palgrave Macmillan Memory Studies.* 2019; pp.151–171. 10.1007/978-3-030-02556-4_8

[ref12] Gray R : Ben Okri’s Eco-Imagination in Every Leaf a Hallelujah (2021): An Afropolitan Approach. *Engl. Acad. Rev.* 2024;41(2):58–69. 10.1080/10131752.2024.2365556

[ref13] Grindheim LT : Beyond uniform reproduction: Exploring children’s imaginative play through the lenses of their teacher. *Contemp. Issues Early Child.* 2020;21(1):58–69. 10.1177/1463949118783384

[ref14] Hajisoteriou C Panaou P Angelides P : Empowering children to speak up and act on social justice issues: The use of folktales and collaborative storytelling as tools of social imagination. *Br. Educ. Res. J.* 2022;48(2):253–271. 10.1002/berj.3765

[ref15] Harris PL : Early Constraints on the Imagination: The Realism of Young Children. *Child Dev.* 2021;92(2):466–483. 10.1111/cdev.13487 33399218

[ref16] Hoff EV : Imagination. *Encyclopedia of Creativity.* Elsevier;2020; pp.617–623. 10.1016/B978-0-12-809324-5.21897-X

[ref17] Howe AJ : Active Imagination and The Legend of Zelda: An Unlikely Source of Modern Mythology. *Psychol. Perspect.* 2022;65(3–4):436–445. 10.1080/00332925.2022.2138198

[ref18] Hutton R : The Wild Hunt in the Modern British Imagination. *Folklore.* 2019;130(2):175–191. 10.1080/0015587X.2018.1493861

[ref19] Jones P Pimenta S Cooke T : *Rewilding Children’s Imaginations: 99 Creative Activities Inspired by Nature and Folktales from Around the World.* London: Routledge;2023. 10.4324/9781003178682

[ref20] Kang H-S : Theoretical possibilities and discussion of Bruner’s narrative curriculum theory. *Information (Japan).* 2017;20(10):7337–7343.

[ref21] Kanik I : Analyses of Food Culture in Kral Şakir Cartoon. *Folklor/Edebiyat.* 2025;31(121):157–178. 10.22559/folklor.2723

[ref22] Lee SC : C. S. Lewis’ mythopoeia of heaven and earth: implications for the ethical and spiritual formation of multicultural young learners. *Int. J. Child. Spiritual.* 2015;20(1):15–28. 10.1080/1364436X.2014.999229

[ref23] Lindqvist G : The dramatic and narrative patterns of play. *Int. J. Phytoremediation.* 2003;11(1):69–78. 10.1080/13502930385209071

[ref24] Majd VK Bakhshaliyev A : The study of psychological aspects of Iranian mothers’ lullabies. *Asian Soc. Sci.* 2016;12(4):93–101. 10.5539/ass.v12n4p93

[ref25] Maksic S Pavlovic Z : Serbian public opinion on child imagination and its correlates. *Zbornik Instituta Za Pedagoska Istrazivanja.* 2008;40(1):7–21. 10.2298/ZIPI0801007M

[ref26] Maksic S Pavlovic Z : Evaluation of child imagination in European cultural-historical context. *Sociologija.* 2009;51(3):263–277. 10.2298/SOC0903263M

[ref27] Monica M Soplantila E : The Role of Local Folklores in Students’ Reading Comprehension. *MATAI: Int. J. Lang. Educ.* 2024;4(2):185–198. 10.30598/matail.v4i2.13717

[ref28] Nurbekov T Salkynbay AB : CulturalAndCognitivePhenomenonIn KazakhFairyTales: AReflexiveEssence. *Folklor/Edebiyat.* 2023;29(116):1179–1196. 10.22559/folklor.2434

[ref29] Oliveira S Cruz M : The gamification octalysis framework within the primary english teaching process: The quest for a transformative classroom. *Rev. Lusofona Educ.* 2018;41(41):63–82. 10.24140/issn.1645-7250.rle41.04

[ref30] Papadaki A Dakouri-Hild A : A Past for/by the Public: Outreach and Reception of Antiquity in Boeotia, Greece. *J. East. Mediterr. Archaeol. Herit. Stud.* 2017;5(3–4):393–410. 10.5325/jeasmedarcherstu.5.3-4.0393

[ref31] Phalafala UP : Polyglot Internationalism and the Matriarchive: The Case of Keorapetse Kgositsile. *Interventions.* 2020;22(3):346–363. 10.1080/1369801X.2020.1718539

[ref32] Pinheiro MA Simão LM : Creativity and Fiction: Interpretative Horizons on the Emergence of the New in the Relationship Between Individual and Culture. *Integr. Psychol. Behav. Sci.* 2021;55(1):1–17. 10.1007/s12124-020-09583-8 33123956

[ref33] Roppola T Whitington V : Pedagogies that engage five to eight year old children’s imagination and creativity at school Tuula Roppola and Victoria Whitington 1 University of South Australia. *J. Educ. Enq.* 2014;13(1):67–81.

[ref34] Rubin L Livesay H : Look, Up in the Sky! Using Superheroes in Play Therapy. *Int. J. Play Ther.* 2006;15(1):117–133. 10.1037/h0088911

[ref35] Rutten K Soetaert R : Narrative and Rhetorical Approaches to Problems of Education. Jerome Bruner and Kenneth Burke Revisited. *Stud. Philos. Educ.* 2012;32(4):327–343. 10.1007/s11217-012-9324-5

[ref36] Sky J : Myths of innocence and imagination: The case of the fairy tale. *Lit. Theol.* 2002;16(4):363–376. 10.1093/litthe/16.4.363

[ref37] St Thomas B Johnson P : Play: The lantern of hope. *J Poetry Ther.* 2004;17(2):81–90. 10.1080/08893670412331296576

[ref38] Stuart I : *Edward Bond: Letters 4.* Routledge;2013. 10.4324/9781315079660

[ref39] Sugara U : Data Systematic Literature Review Folklore.[Data set].2026. 10.5281/zenodo.20034230

[ref40] Uǧurlu EK : Lullaby as cultural memory transmitter. *Milli Folklor.* 2014;26(102):43–52.

[ref41] Van der Riet P Jitsacorn C Junlapeeya P : Nurses’ stories of a “Fairy Garden” healing haven for sick children. *J. Clin. Nurs.* 2014;23(23–24):3544–3554. 10.1111/jocn.12637 24899398

[ref42] Walker S : Young people’s mental health: The spiritual power of fairy stories, myths and legends. *Ment. Health Relig. Cult.* 2010;13(1):81–92. 10.1080/13674670903196721

[ref43] Woolley JD Gilpin AT : Development of Imagination and Fantasy. *Encyclopedia of Infant and Early Childhood Development.* Elsevier;2020; pp.430–437. 10.1016/B978-0-12-809324-5.21821-X

[ref44] Woolley JD Tullos A : Imagination and Fantasy. *Encyclopedia of Infant and Early Childhood Development.* Elsevier;2008; pp.117–127. 10.1016/B978-012370877-9.00081-5

