# Supplementary material for: How can folklore build children’s imagination: A systematic literature review
Source: F1000Res. 2026 Jun 15;15:928. [Version 1] doi: 10.12688/f1000research.180332.1 (PMC13562922; doi:10.12688/f1000research.180332.1)
Supplement: Peer review history

# Reviewer report: Reviewer response for version 1

**DOI:** 10.5256/f1000research.198934.r507570

**Published:** 2026-09-02

**Authors:** Hery Nuraini

**Affiliations:** 1 English Education Department Institution, Universitas Islam Syekh-Yusuf, Tangerang, Banten, Indonesia

Recomendation: Minor Revision

 Overall this manuscript has a pontential contribution in education field. Please add more citations from the previous study or theories in part of introduction and discussion the finding. 

 The manuscript addressed a worthwhile topic and good publication potential. But the authors should be elaborated the three issues:

 1. clarity the conceptual boundaries of folklore and imagination

 2. verify that all 23 included studies genuinely meet the inclusion criteria

 3. restructure the results and discussions according to RQ1 - RQ4

Are the rationale for, and objectives of, the Systematic Review clearly stated?

Yes

Is the statistical analysis and its interpretation appropriate?

Yes

If this is a Living Systematic Review, is the ‘living’ method appropriate and is the search schedule clearly defined and justified? (‘Living Systematic Review’ or a variation of this term should be included in the title.)

Partly

Are sufficient details of the methods and analysis provided to allow replication by others?

Yes

Are the conclusions drawn adequately supported by the results presented in the review?

Partly

Reviewer Expertise:

English Education, Local Wisdom

I confirm that I have read this submission and believe that I have an appropriate level of expertise to confirm that it is of an acceptable scientific standard.

# Reviewer report: Reviewer response for version 1

**DOI:** 10.5256/f1000research.198934.r507567

**Published:** 2026-08-24

**Authors:** Onok Yayang Pamungkas

**Affiliations:** 1 Universitas Muhammadiyah Purwokerto, Kembaran, Indonesia

Approval Status: Not Approved

 The topic is relevant and the four research questions are clearly stated. However, the manuscript cannot yet be considered a valid systematic review because the included evidence does not consistently follow the authors’ own eligibility criteria. Substantial methodological reconstruction is required.

 Major Comments

**1. The included studies do not match the eligibility criteria**

 Table 1 restricts inclusion to empirical studies involving primary-school students. However, Table 3 includes literary analyses, conceptual papers, clinical discussions, preschool studies, and publications without direct child participants. Examples include Lee (2015), Gray (2024), Hutton (2019), Walker (2010), and Sky (2002). The authors should repeat the eligibility assessment and exclude studies that do not meet the stated population and design criteria. Alternatively, the manuscript should be redesigned as a broader scoping review, with corresponding changes to its objectives and conclusions.

**2. The screening process is internally inconsistent**

 The PRISMA flow reports duplicates at more than one stage, states that no reports were not retrieved, but later excludes articles because the full text was unavailable. “Discontinued journal” is also used as an exclusion reason although it is not listed in Table 1 and is not a methodological reason to exclude an eligible study. The flow diagram should be reconstructed from the original screening records, with a citation-level list of full-text exclusions.

**3. The review is not reproducible**

 The manuscript does not provide database-specific search syntax, searched fields, deduplication procedures, number of reviewers, disagreement resolution, data-extraction procedures, or coding methods. It is therefore unclear how the categories in the “Aspect” column of Table 3 were developed. The authors should provide the complete search strategies, extraction form, coding framework, and study-to-theme matrix.

**4. No methodological quality assessment is reported**

 Journal quartiles and citation counts in Table 2 do not indicate the quality or risk of bias of individual studies. This is particularly important because the evidence ranges from classroom research to literary interpretation and clinical opinion. An appropriate study-level appraisal should be conducted, and its results should inform the synthesis.

**5. The conclusions overstate the evidence**

 Terms such as “significantly,” “highly effective,” and “has proven effective” are not supported by statistical synthesis or comparative evidence. For example, the Fairy Garden findings are based on nurses’ interpretations rather than direct child data, while the gamification claim relies primarily on a small qualitative study. These statements should be revised to distinguish observed outcomes, participant reports, third-party interpretations, and theoretical propositions.

**6. Data and AI-assisted procedures require greater transparency**

 The repository should include database exports, screening decisions, full-text exclusion reasons, extraction data, coding categories, and quality-appraisal results. The use of NotebookLM and Rayyan AI for screening and preliminary categorisation should also be described in the Methods, including the extent of human verification.

 A final audit is also necessary. The statement concerning children in “communist or religious countries” is overly generalised and should be removed. The phrase “most recent decade” is inaccurate for the period 2000–2025, “folktales” is recommended as a future keyword even though it already appears in the search string, and the journal information for Lindqvist (2003) appears incorrect.

**Conclusion**

 The manuscript addresses an important subject, but its present corpus, screening process, and synthesis do not support the reported conclusions. The review should be rerun using consistent eligibility criteria, transparent screening records, formal quality appraisal, and evidence-calibrated claims.

Are the rationale for, and objectives of, the Systematic Review clearly stated?

Partly

Is the statistical analysis and its interpretation appropriate?

Not applicable

If this is a Living Systematic Review, is the ‘living’ method appropriate and is the search schedule clearly defined and justified? (‘Living Systematic Review’ or a variation of this term should be included in the title.)

Not applicable

Are sufficient details of the methods and analysis provided to allow replication by others?

No

Are the conclusions drawn adequately supported by the results presented in the review?

No

Reviewer Expertise:

children literature, folklore, edication

I confirm that I have read this submission and believe that I have an appropriate level of expertise to state that I do not consider it to be of an acceptable scientific standard, for reasons outlined above.

# Reviewer report: Reviewer response for version 1

**DOI:** 10.5256/f1000research.198934.r507574

**Published:** 2026-08-19

**Authors:** Zulfa Fahmy

**Affiliations:** 1 Universitas Islam Negeri Walisongo Semarang, Semarang, Central Java, Indonesia

This study presents a systematic literature review (SLR) following PRISMA 2020 guidelines to examine how folklore fosters elementary school children’s imagination. Searching Scopus and Web of Science for articles published between 2000 and 2025, the authors screened 189 records, reducing them to 23 core empirical studies. The qualitative synthesis addresses four main research questions: (1) folklore forms used (folktales, myths, lullabies, modern mythology), (2) effective pedagogical strategies (read-alouds with physical manipulatives, gamification, collaborative storytelling), (3) impacted aspects of imagination (identity formation, social imagination/empathy, active imagination), and (4) existing research gaps in the literature.

 The manuscript is clear, well-structured, and methodologically transparent. Below is a detailed breakdown expanding on the review criteria:

1. **Rationale and Objectives**
  The rationale is well-constructed: the authors clearly demonstrate that the existing literature on folklore and imagination remains fragmented and that current school curricula tend to prioritize standardized competencies over imaginative development. The four Research Questions (RQs) are well-articulated and directly guide the subsequent thematic analysis.
2. **Methods and Reproducibility **
  The methodology is robust and fully reproducible. The explicit mention of search strings, databases (Scopus and Web of Science), date range (2000–2025), search execution timestamp (November 27, 2025, at 12:40 WIB), inclusion/exclusion criteria, and the PRISMA flow diagram provide complete transparency.
3. **Statistical Analysis and Interpretation **
  As a qualitative systematic review, no quantitative meta-analysis or statistical hypothesis testing was performed. The data processing relies on thematic categorization, which is appropriate to the synthesis's descriptive nature.
4. **Conclusions and Evidence**
  The conclusions align well with the qualitative synthesis. The primary assertion, that folklore serves as a powerful pedagogical tool for stimulating cognitive, affective, and social imagination, is thoroughly supported by the synthesis of the 23 reviewed studies.
5. **Living Systematic Review Status**
  This manuscript is a standard, single-instance systematic review and does not claim to be a Living Systematic Review.

While the manuscript is methodologically sound and ready for publication in its current form, the authors could further strengthen the work by addressing the following minor enhancements:

- **Methodological Risk of Bias Assessment:** Include an explicit critical appraisal or quality assessment tool (e.g., MMAT or CASP) for the 23 included studies to assess potential methodological bias across the synthesized literature.
- **Expanded Discussion on Limitations:** Elaborate slightly on the potential selection bias introduced by restricting the search strictly to English-language publications, as folklore research often appears in regional non-English journals.

Are the rationale for, and objectives of, the Systematic Review clearly stated?

Yes

Is the statistical analysis and its interpretation appropriate?

Not applicable

If this is a Living Systematic Review, is the ‘living’ method appropriate and is the search schedule clearly defined and justified? (‘Living Systematic Review’ or a variation of this term should be included in the title.)

Not applicable

Are sufficient details of the methods and analysis provided to allow replication by others?

Yes

Are the conclusions drawn adequately supported by the results presented in the review?

Yes

Reviewer Expertise:

Cultural studies, Folklore Studies, Indonesian Literature

I confirm that I have read this submission and believe that I have an appropriate level of expertise to confirm that it is of an acceptable scientific standard.
